# Effect of postoperative coffee consumption on gastrointestinal function after abdominal surgery: A systematic review and meta-analysis of randomized controlled trials

**DOI:** 10.1038/s41598-018-35752-2

**Published:** 2018-11-26

**Authors:** Nuntasiri Eamudomkarn, Chumnan Kietpeerakool, Srinaree Kaewrudee, Nampet Jampathong, Chetta Ngamjarus, Pisake Lumbiganon

**Affiliations:** 1 Department of Obstetrics and Gynaecology, Faculty of Medicine, Khon Kaen University, Khon Kaen, Thailand; 2 Department of Epidemiology and Biostatistics, Faculty of Public Health, Khon Kaen University, Khon Kaen, Thailand

## Abstract

Coffee is believed to prevent postoperative ileus. This systematic review and meta-analysis was undertaken to determine the effectiveness of coffee consumption in stimulating gastrointestinal function after abdominal surgery. A number of databases for randomized controlled trials comparing coffee consumption following abdominal surgery versus water drinking or no intervention were searched. Cochrane’s Risk of Bias tool was used to assess risk of bias in included studies. Six trials involving 601 participants were included. All studies had high risk of performance bias. Three studies had an unclear risk of selection bias. Postoperative coffee consumption reduced time to first defecation (mean difference (MD), −9.98 hours; 95% CI, −16.97 to −2.99), time to first flatus (MD, −7.14 hours; 95% CI, −10.96 to −3.33), time to first bowel sound (MD, −4.17 hours; 95% CI, −7.88 to −0.47), time to tolerance of solid food (MD, −15.55 hours; 95% CI, −22.83 to −8.27), and length of hospital stay (MD, −0.74 days; 95% CI, −1.14 to −0.33). Benefits increased with increasing complexity of the procedure. None of the included studies reported adverse events associated with coffee consumption. Postoperative coffee consumption is effective and safe for enhancing the recovery of gastrointestinal function after abdominal surgery.

## Introduction

Although surgical techniques and perioperative care have improved with time, postoperative ileus (POI) remains a frequent complication following abdominal surgery^1^. POI is a transient impairment of coordinated bowel motility in response to the trauma of surgery thus leading to accumulation of gastrointestinal secretions within the lumen of the gut^1,2^. Symptoms of POI include failure to pass stool or flatus, nausea, vomiting, and abdominal pain and usually lasts 2 to 4 days after surgery^1^. Factors associated with an increased risk of POI following abdominal surgery include colorectal surgery, open abdominal surgery, emergency operations, prolonged operative time, amount of opioid analgesic use, longer nasogastric catheter use, and smoking history^1,3,4^. POI contributes to delay patient recovery, prolonged hospital stay, and increased post-operative morbidity and healthcare costs^1,5^.

The pathophysiology of postoperative ileus is multifactorial^1,2^. There are three proposed mechanisms involved in the development of POI, namely neurogenic, inflammatory and pharmacological mechanisms. The neurogenic mechanisms contribute to the immediate phase of POI occurring during operation by inhibiting sympathetic stimulation and increasing adrenergic motor neuronal activity. The second phase of POI, which typically occurs 3–4 h after operation, is mediated through the inflammation process caused by the release of pro-inflammatory cytokines and chemokines. This inflammatory response then activates the phagocytes residing throughout the gut to release nitric oxide and prostaglandins which reduce bowel mobility by inhibiting smooth muscle contractility^1,2^. Several pharmacological agents can diminish gastrointestinal peristalsis. The use of a crystalloid solution can result in intestinal edema and stretching. Common anesthetic agents such as halothane, enflurance, and atropine prolong gastric emptying times which increase risks of postoperative nausea and vomiting. Postoperative narcotic analgesia is associated with decreased gastrointestinal mobility by activating opioid receptors^1,2^.

Coffee is one of the most popular beverages consumed worldwide. Coffee consists of several biologically active compounds, such as caffeine, diterpenes, and chlorogenic acids, which can positively affect human health^6^. Coffee consumption mitigates the risks of cardiovascular and metabolic disorders by its antioxidant activity^6^. Coffee is associated with a reduction in the incidence of diabetes, liver disease, and Parkinson’s disease^6^. In addition, coffee consumption trends to reduce mortality^6^.

Coffee also induces bowel movement^7,8^. Coffee stimulates motor activity of the large intestine within a few minutes after intake, particularly in the transverse and descending colon^7,8^. Based on this finding as well as its popularity as beverage and low cost, coffee might be a promising option to hasten the recovery of gastrointestinal function after abdominal surgery. Therefore, it is crucial to establish strong evidence as to whether postoperative coffee consumption is effective and safe for alleviating POI. Accordingly, this systematic review and meta-analyses of randomized controlled trials was conducted to determine the influence of postoperative coffee consumption in enhancing the early recovery of gastrointestinal function following abdominal surgery.

## Methods

The details of the protocol for this systematic review were registered with PROSPERO **(**CRD42018085090). This meta-analysis was performed and reported according to the PRISMA statement^9^.

### Criteria for considering studies for this review

Randomized controlled trials (RCTs) comparing coffee consumption following any kind of abdominal surgery versus drinking water or no intervention irrespective of language of publication, publication status, year of publication, or sample size were included.

### Types of outcome measures

The primary outcome was the time to first defecation. Secondary outcomes included the time to first passage of flatus, the time to first bowel movement sound, the time to tolerance of solid food, possible adverse effects of postoperative coffee intake, and length of hospital stay.

### Search methods for identification of studies

To identify potential eligible studies, a systematic literature search was conducted using the major electronic databases including MEDLINE, Scopus, ISI Web of Science, Pubmed, CINAHL, and Cochrane Central Register of Controlled Trials (CENTRAL) from their inception to March 2018. Reference lists of articles were retrieved by the search and authors of the trials contacted to obtain additional data if necessary. In addition, ClinicalTrials.gov and the WHO International Clinical Trials Registry Platform (www.who.int/ictrp) were searched for unpublished, planned and ongoing trial reports. Open Grey (www.opengrey.eu) was searched for grey literature. To ensure the comprehensive searches, the titles of all relevant articles were identified on Google Scholar and then a further search was made related to these studies focusing on the first 50 records identified^10^.

### Study selection and data extraction

Titles and abstracts of studies retrieved by electronic searching were screened independently by two review authors. Those studies where their titles and abstracts clearly did not meet the inclusion criteria were excluded. The full texts of potentially eligible studies were retrieved and independently assessed by two review authors. Figure I presents the PRISMA flow diagram. The risk of bias of included studies was independently evaluated by two authors using the Cochrane Risk of Bias Tool for Randomized Controlled Trials^11^. Data were extracted independently onto a data abstraction form specifically designed for the review. Any disagreements were resolved through discussion with a third person.

### Statistical analysis

Statistical analysis using the Cochrane Review Manager (RevMan) software was performed^12^. The random-effects model with inverse variance weighting for all meta-analyses as between-study heterogeneity that was expected was applied^13,14^. For continuous outcomes, e.g. time to first defecation, time to first flatus, time to first bowel sound, time to tolerance of solid food, and length of hospital stay, the mean differences (MD) with their 95% confidence intervals (CI) were calculated. If continuous outcomes were expressed as median and range, the study author was contacted to obtain sample mean and standard deviation (SD) data. If this was not possible, the data were converted to mean and SD using the formulae proposed by Wan *et al*.^15^. For dichotomous outcomes (postoperative nausea), the risk ratios (RR) and the 95% CI were calculated.

Statistical heterogeneity in each meta-analysis using the I² statistic was assessed: values of I^2^ greater than 50% were indicative of significant heterogeneity and the Chi² tests, where a cut off of p value < 0.1 indicating whether there was statistical evidence of heterogeneity were considered^14^. Subgroup analysis was carried out according to the types of operative procedure. Sensitivity analyses to assess the robustness of the findings by repeating the analysis excluding studies judged to be at high risk or unclear risk of selection bias was performed. A leave-one-out sensitivity analysis was also performed by iteratively excluding one included study with largest effect size to confirm that our findings were not driven by any single study.

## Results

### Characteristics of included studies

**Figure 1:**
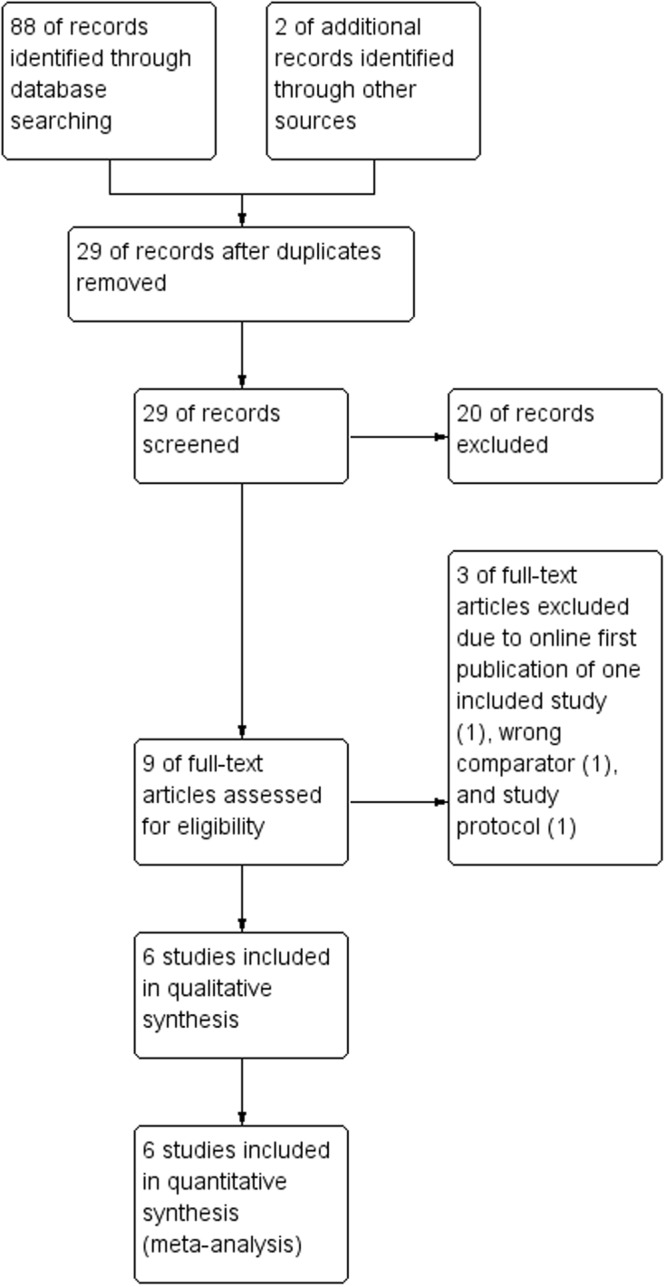
PRISMA flow-diagram of literature search and study selection.

A broad search yielded 88 references from the electronic database searches. Two additional references from other sources were identified. After de-duplication, 29 references were screened and 20 references were excluded that obviously did not meet the inclusion criteria. Of the nine studies that potentially met the review inclusion, six studies were included after reviewing the full texts involving 601 participants (Supplementary Table S1). Figure 1 displays the PRISMA flowchart for study selection. Five included studies published in peer-reviewed journals^16–20^. One included study was only available in an academic search engine (Supplementary Table S1).

Of the six included studies, a study of Dulskas *et al*.^18^ divided the participants into three groups to receive regular coffee, decaffeinated coffee, and water. Participants who received two different kinds of coffee in this included study were then combined into a single group to create a single pair-wise comparison. All participants in a study of Göymen *et al*.^17^ received decaffeinated coffee. The remaining four included studies used regular coffee. Göymen *et al*.^17^ reported their continuous outcomes as median and interquartile range. Two groups of participants in a study of Göymen *et al*.^17^ who received no intervention or water were combined into a single control group.

Of the six included studies, three evaluated the effects of coffee consumption after cesarean delivery. The remaining three included studies were undertaken among participants undergoing colorectal (2 studies) and gynecologic cancer surgery (1 study). Of the two included studies that were undertaken among participants undergoing colorectal surgery, one study evaluated the effects of coffee consumption following laparoscopic left-sided colectomy. Approximately 61% of participants in the remaining study underwent laparotomy for colectomy. All participants in the study of gynecologic cancer surgery underwent the laparotomy approach. The operations performed in all included studies were elective procedures (Supplementary Table S1).

All of studies included in this review do not clearly state the use of the Enhanced Recovery after Surgery (ERAS) protocol or other ‘fast-track’ protocols during postoperative care.

Four ongoing trials which are determining the coffee consumption after small or large bowel resections were identified (Supplementary Table S2).

### Risk of bias in included studies

**Figure 2:**
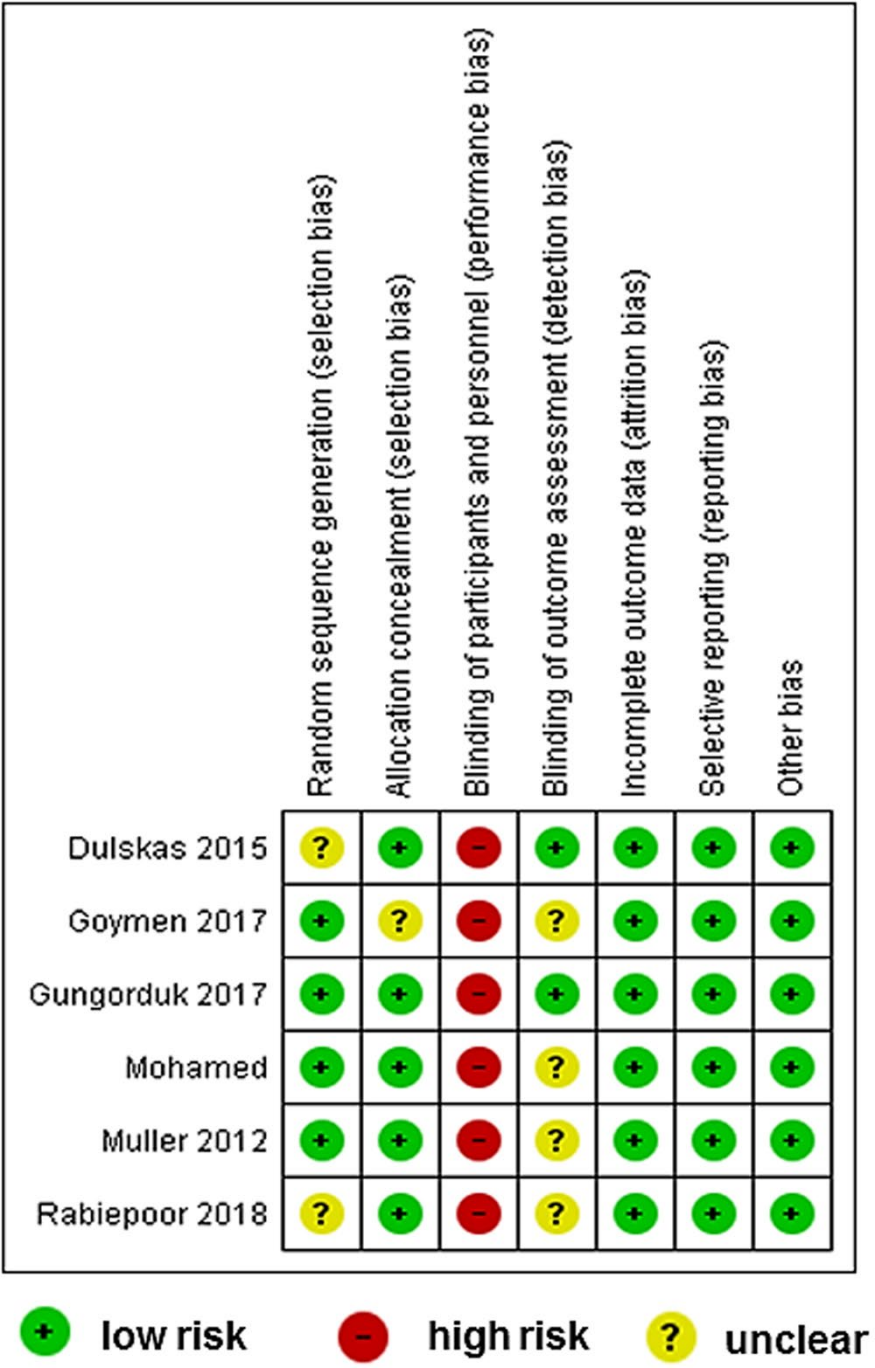
Summary of the risk of bias for each included study using the Cochrane Risk of Bias Tool for Randomized Controlled Trials.

Figure 2 presents the summary of the risk of bias for each included study. All included studies had high risk of performance bias as they were unable to blind the participants about the intervention allocated. Three included studies were determined as to be of unclear risk of selection bias (Supplementary Table S1).

### Effects of interventions

#### Time to first defecation

**Figure 3:**
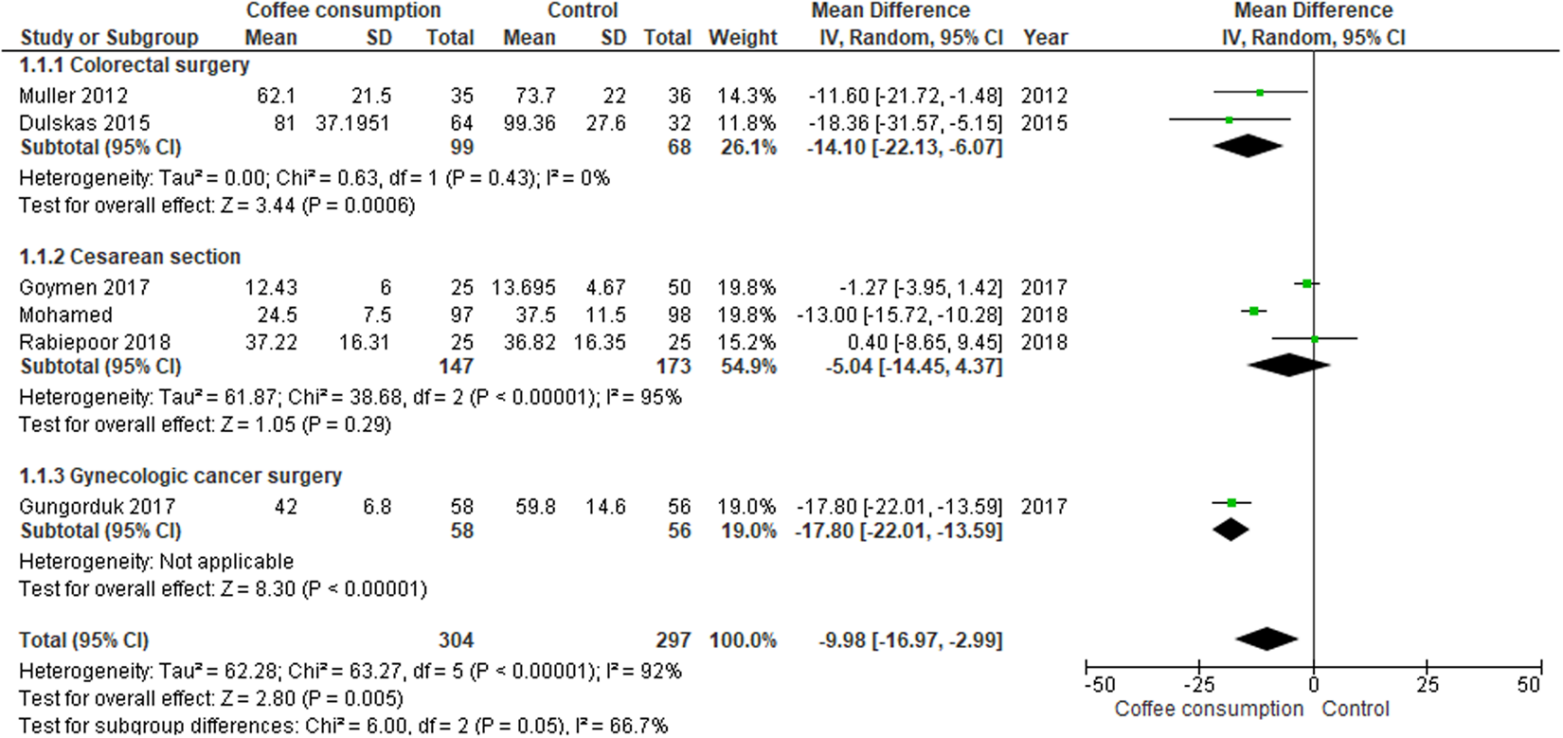
Forest plot of mean differences with corresponding 95% CIs of studies on time to first defecation (hours) by operation types.

Meta-analyses assessing 601 participants indicated that postoperative coffee consumption reduced the time to first defecation when compared to water or no intervention (MD, −9.98 hours; 95% CI, −16.97 to −2.99 hours). Subgroup analysis, however, revealed that the significant effect of coffee consumption in reducing the time to first defecation was observed in either colorectal or gynecologic cancer surgeries but not in cesarean delivery (Fig. 3).

#### Time to first flatus

**Figure 4:**
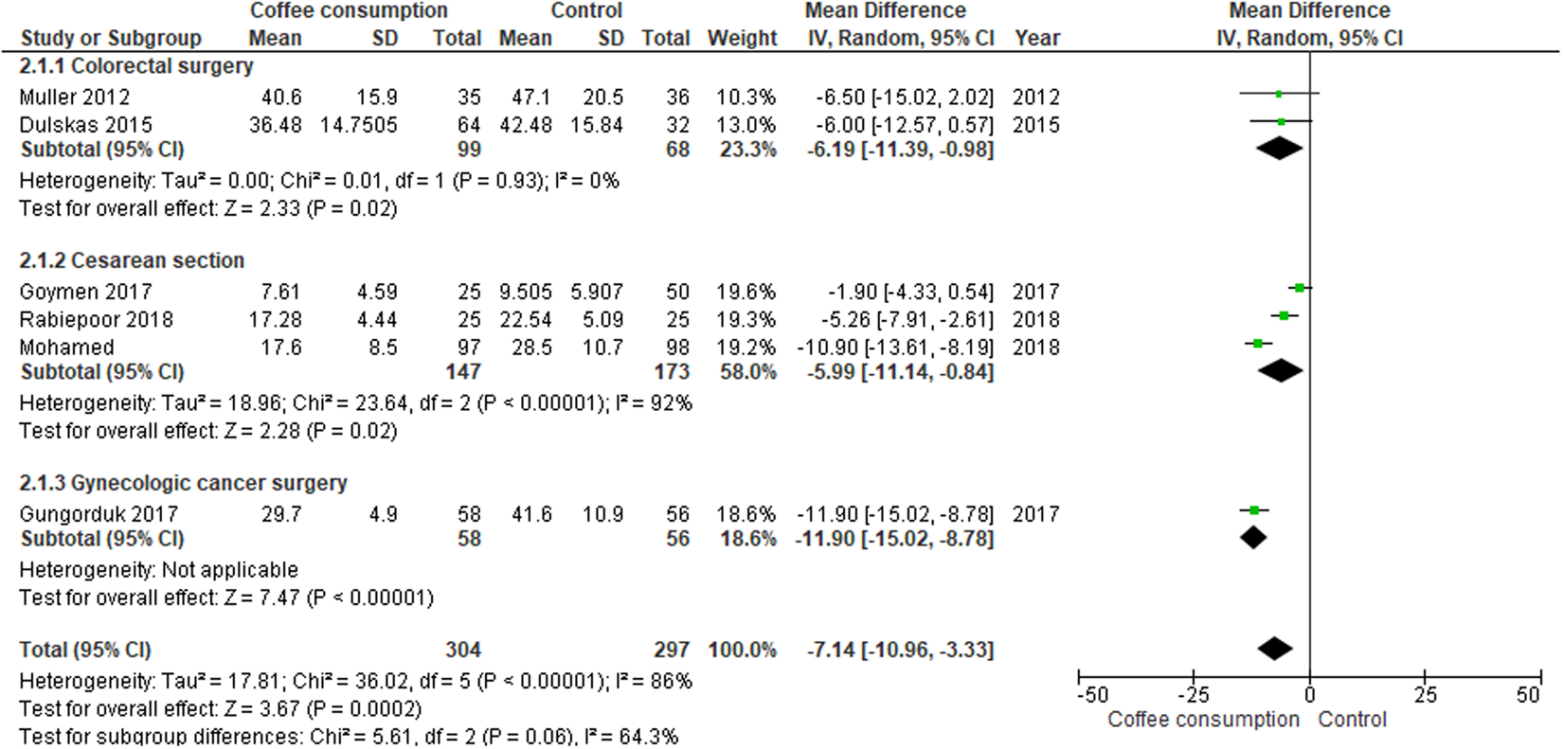
Forest plot of mean differences with corresponding 95% CIs of studies on time to first flatus (hours) by operation types.

Coffee consumption after operation significantly decreased the time to first flatus when compared to water or no intervention (MD, −7.14 hours; 95% CI, −10.96 to −3.33 hours). The significant impact of coffee in reducing the time to first flatus was observed across the three different types of surgical procedures, with the largest magnitude of effect in participants undergoing gynecologic cancer surgery (Fig. 4).

#### Time to first bowel sound

**Figure 5:**
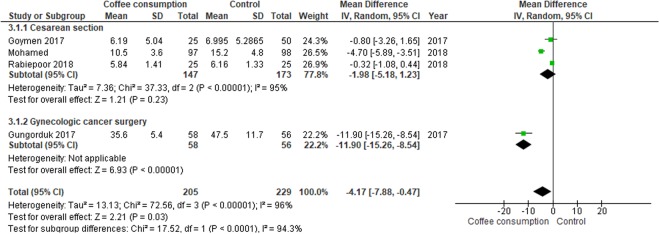
Forest plot of mean differences with corresponding 95% CIs of studies on time to first bowel sound (hours) by operation types.

The time to the first audible bowel sound was only evaluated in 434 participants who underwent cesarean delivery or gynecologic cancer surgery. Overall, participants allocated to the coffee group had a shorter time to first audible bowel sound when compared to those in the control group (MD, −4.17 hours; 95% CI, −7.88 to −0.47 hours). In subgroup analysis, however, the significant effect was solely observed among participants undergoing gynecologic cancer surgery (Fig. 5).

#### Time to tolerance of solid food

**Figure 6:**
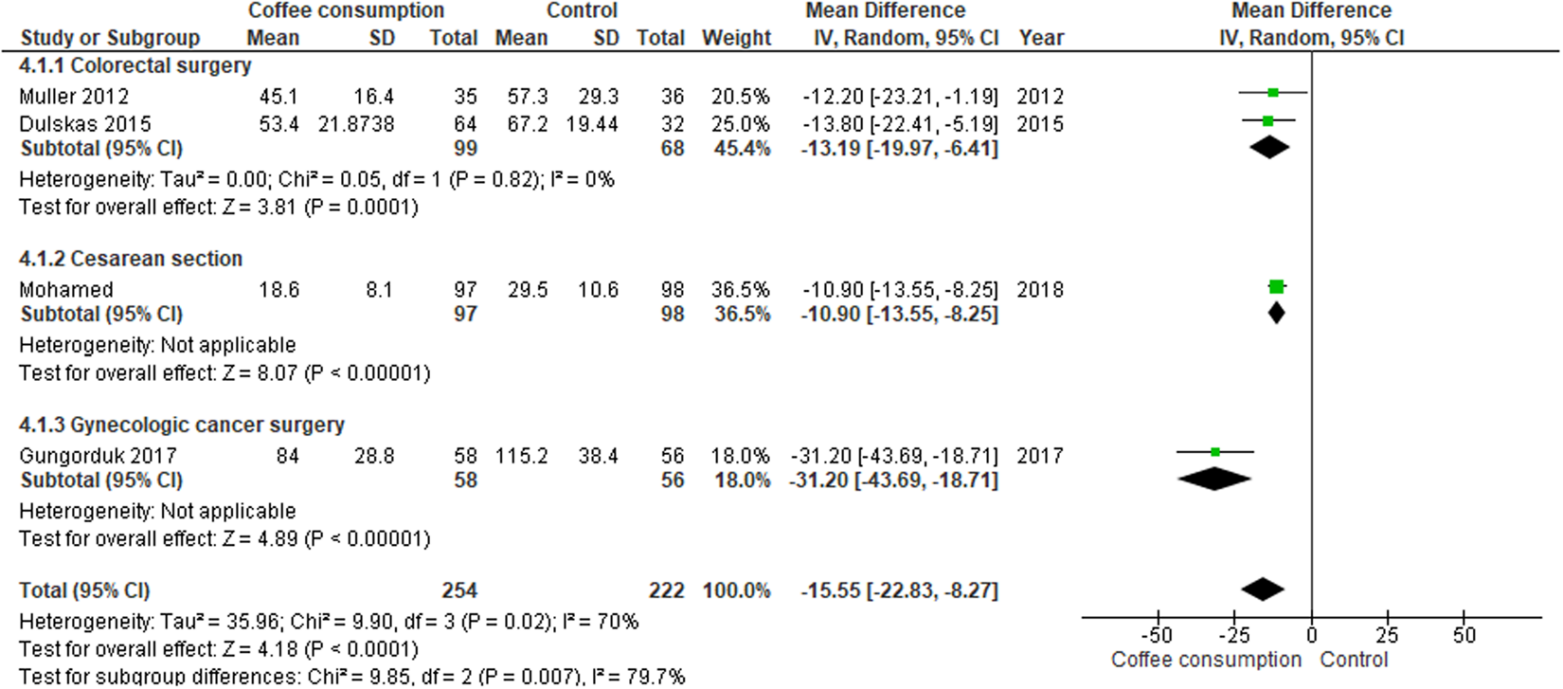
Forest plot of mean differences with corresponding 95% CIs of studies on time to tolerance of solid food (hours), by operation types.

Meta-analysis assessing 476 participants showed that participants with coffee consumption had a shorter time to tolerance of solid food than those given water or no intervention (MD, −15.55 hours; 95% CI, −22.83 to −8.27 hours). The significant shorter time to tolerance of solid food after coffee consumption was noted across the three different types of operations (Fig. 6).

#### Postoperative nausea

**Figure 7:**
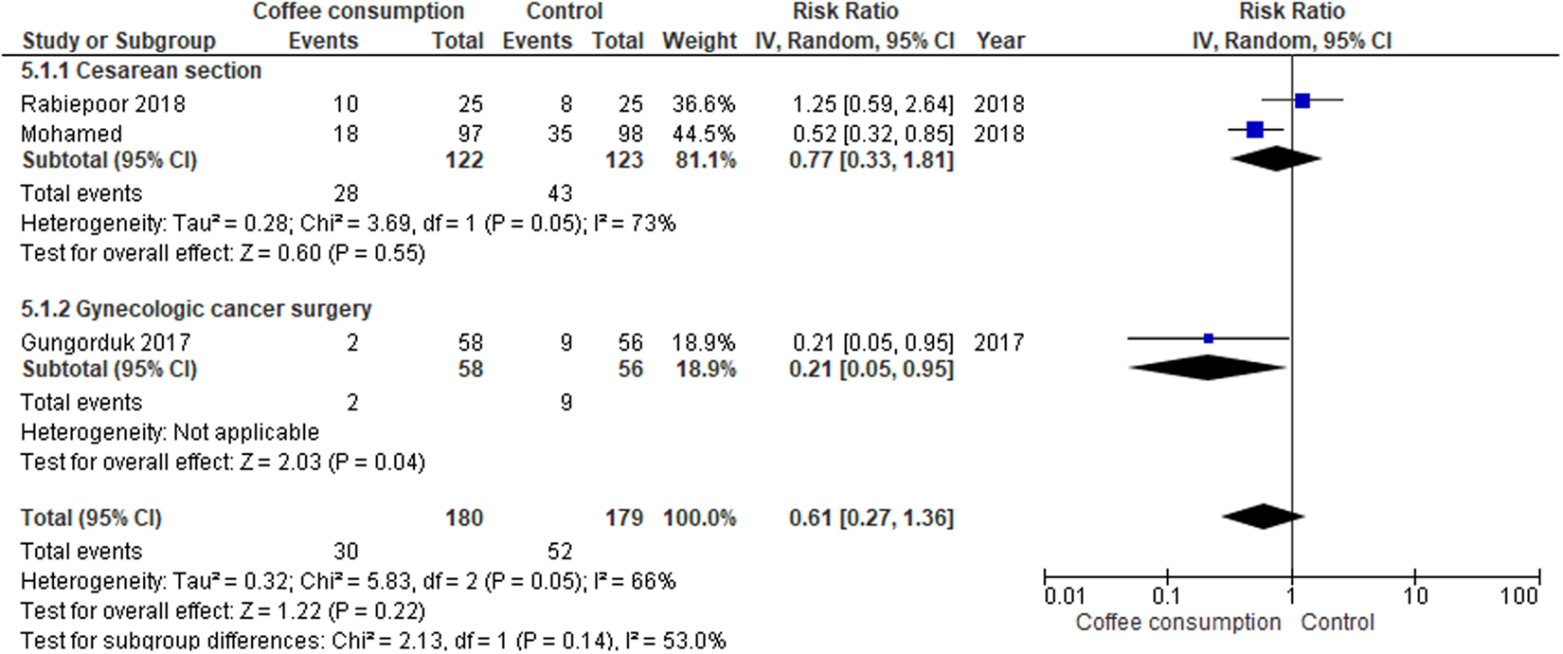
Forest plot of risk ratios with corresponding 95% CIs of studies on postoperative nausea by operation types.

Postoperative nausea was assessed in 359 participants undergoing cesarean delivery and gynecologic cancer surgery (Fig. 7). Overall, there was no significant difference in the risk of postoperative nausea between the participants assigned to the coffee group and those in the control group (RR, 0.61; 95% CI, 0.27 to 1.36). There was, however, a significantly lower risk of postoperative nausea in participants undergoing gynecologic cancer surgery who received coffee than that in the control group (RR, 0.21; 95% CI, 0.05 to 0.95).

#### Length of hospital stay

**Figure 8:**
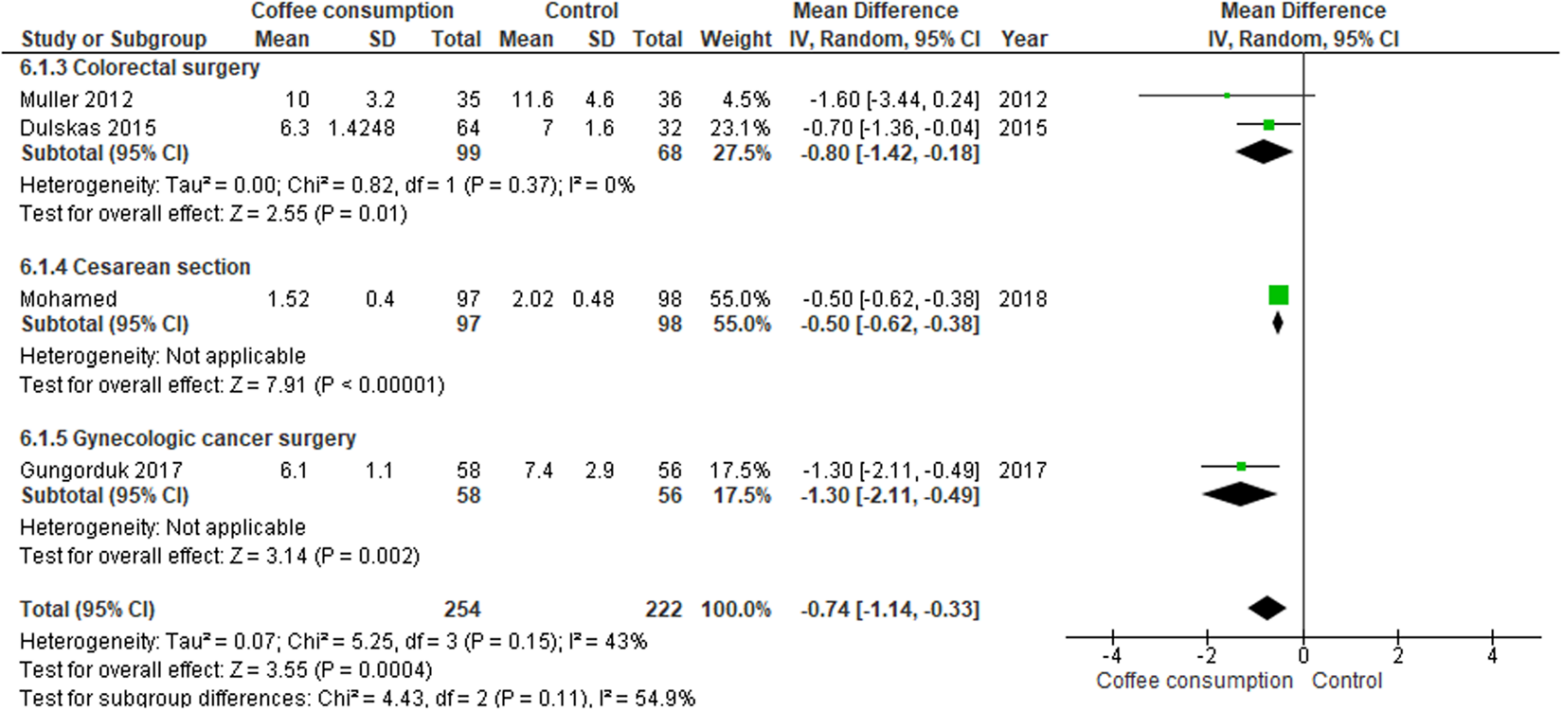
Forest plot of mean differences with corresponding 95% CIs of studies on length of hospital stay (days) by operation types.

Meta-analysis assessing 476 participants indicated a shorter length of hospital stay among the participants in the coffee group than those assigned to the control group (MD, −0.74 days; 95% CI, −1.14 to −0.33 days), with the largest benefit in gynecologic cancer surgery (MD, −1.30; 95% CI, −2.11 to −0.49 days; Fig. 8).

#### Sensitivity analysis

After excluding three studies that were judged to have an unclear risk of selection bias^17,18,20^, the magnitude of favorable effects of postoperative coffee consumption in shortening the time to first defecation, time to first flatus, and time to first audible bowel sounds was substantially increased with a more precise estimate of their corresponding 95% CIs. The overall effects on the time to tolerance of solid food, postoperative nausea, and length of hospital stay, however, tended to be unchanged (Table 1). Leave-one-out analyses which removed a study of Gungorduk *et al*.^19^ showed no marked difference in the results excepting time to first bowel sound movement which may indicate that the most of the pooled estimates were not solely driven by one single study (Table 1).

**Table 1:** Sensitivity analysis.

Outcomes	Overall results	Sensitivity analysis: I	Sensitivity analysis: II
Time to first defecation (MD; hours)	−9.98 (95% CI, −16.97, −2.99)	−14.63 (95% CI, −18.33, −10.92)	−8.11 (95% CI, −15.58, −0.63)
Time to first flatus (MD; hours)	−7.14 (95% CI, −10.96, −3.33)	− 11.04 (95% CI, −13.02, −9.06)	−6.05 (95% CI, −9.97, −2.14)
Time to first bowel sound movement (MD; hours)	−4.17 (95% CI, −7.88, −0.47)	−8.12 (95% CI, −15.17, −1.07)	−1.98 (95% CI, −5.18, 1.23)
Time to tolerance of solid food (MD; hours)	−15.55 (95% CI, −22.83, −8.27)	−16.93 (95% CI, −27.97, −5.89)	−11.20 (95% CI, −13.67, −8.76)
Postoperative nausea (RR)	0.61 (95% CI, 0.27, 1.36)	0.45 (95% CI, 0.23, 0.86)	0.77 (95% CI, 0.33,1.81)
Length of hospital stay (MD; days)	−0.74 (95% CI, −1.14, −0.33)	−0.88 (95% CI, −1.58, −0.19)	−0.51 (95% CI, −0.63, −0.39)

Abbreviation: MD, mean difference; CI, confidence interval; RR, risk ratio.

Sensitivity analysis I: Excluding studies judged to be at unclear risk of selection bias.

Sensitivity analysis II: Leave-one-out analysis excluding one study with largest effect size.

## Discussion

### Main findings

Evidence from this review is based on six RCTs with 601 participants. It was found that postoperative coffee consumption significantly reduced the time to first defecation, time to first flatus, time to first audible bowel sound, and time to tolerance of solid food. Postoperative coffee consumption slightly reduced the length of hospital stay. The effect sizes appeared to be directly associated with the complexity of surgical procedures with the largest benefit in gynecologic cancer surgery and smallest in cesarean delivery. However, the results observed in the patients with gynecologic cancer were based on a single small study, future RCTs with a sufficiently sample size is required to confirm these promising results.

Sensitivity analyses applying risk of selection bias and leave-one out basis showed no clinically important changes in the findings. None of the six included studies reported adverse events associated with coffee consumption.

### Strengths and limitations of the study

This systematic review has some strengths. First, investigators are not aware of any previous systematic reviews determining the effectiveness of postoperative coffee consumption with the aim of enhancing the recovery of gastrointestinal functions following surgery. Second, this review restricted included studies to RCTs in order to use only the strongest evidence. Third, this systematic review indicates the benefits of coffee consumption in promoting the recovery of gastrointestinal functions that were observed across different types of abdominal surgeries. In addition, the robustness of the review findings was reaffirmed by sensitivity analyses.

This review has some limitations. As it was not feasible to blind the participant to intervention received, all included studies were rated at high risk of performance bias which may influence some treatment effect estimates. The operations performed in all included studies were elective procedures. Therefore, generalization of the results to the patients undergoing emergency operations may be limited. A limited number of included studies that where minimally invasive surgery was performed precluded the ability to carry out a subgroup analyses to assess the impact of the types of surgical approaches.

### Interpretation in light of other evidence

Controversy still exists about whether the effects of decaffeinated coffee on the recovery of gastrointestinal functions are comparable to regular coffee. In one of the included studies that was conducted among women undergoing cesarean delivery, all participants allocated in the intervention group received decaffeinated coffee. The findings of this study found no significant differences in the recovery of gastrointestinal functions after decaffeinated coffee consumption in terms of time to first defecation (MD, −1.27 hours; 95% CI, −3.95 to 1.42), time to first flatus (MD, −1.90 hours; 95% CI, −4.33 to 0.54), and time to first audible bowel sound (MD, −0.80; 95% CI, −3.26 to 1.65) when compared to the control group^17^. In contrast, another included study in which two of the three groups of participants received regular coffee (28 participants) and the third decaffeinated coffee (28 participants) showed comparable benefits in stimulating gastrointestinal functions following colorectal surgery between these two comparison groups in terms of the time to defecation (3.75 versus 3.00 hours), time to first flatus (1.57 versus 1.47 hours), and time to tolerance of solid food (2.60 versus 1.85 hours)^18^. These available data were too sparse to permit drawing meaningful conclusions.

Although there were no reported adverse events associated with postoperative coffee consumption in any included study in this review, coffee is not without potential adverse effects; for instance, coffee drinking acutely raises blood pressure and heart rate which are secondary to an increased circulating concentrations of norepinephrine^21,22^.Thus hypertensive patients may be discouraged from postoperative coffee consumption^22^. In addition, coffee drinking may have adverse sleep-related consequences including decreased total sleep time, difficulty falling asleep, increased nocturnal awakenings, and daytime sleepiness^23,24^. Although while the negative impact of coffee on sleep patterns in healthy individuals are mostly mild, it may lead to increased worrying and complicate the well-being in postoperative patients.

Chewing gum is another promising intervention for enhancing the early recovery of gastrointestinal functions after operation. The Cochrane systematic review assessing the effectiveness of chewing gum after surgery reported some evidence for the benefit of postoperative chewing gum in improving recovery of gastrointestinal functions by reduction of time to first flatus (−10.4 hours; 95% CI: −11.9, −8.9), time to bowel movement (−12.7 hours; 95% CI: −14.5, −10.9), time to first bowel sound (−5.0 hours; 95% CI: −6.4, −3.7). In addition, use of chewing gum slightly reduced length of hospital stay (−0.7 days; 95% CI: −0.8, −0.5)^25^. Similar to the present findings, the effect sizes of intervention on outcomes were directly related to the complexity of the surgical procedure. The benefits were substantial in colorectal surgery but only minimal in cesarean delivery^25^. Interestingly, this review suggested that the effect of chewing gum on outcomes was generally reduced in the analysis of studies conducted within an ERAS context^25^.

### Implications for review findings

This present review highlights that postoperative coffee consumption is an innovative intervention to hasten the recovery of gastrointestinal function following abdominal surgery given that it is generally well tolerated by individuals, low-cost, widely available and easy to implement. Based on these promising results, clinicians may consider advising patients to drink coffee during postoperation period as appropriate.

More studies are needed to explore the effects of coffee consumption after a variety of different surgical settings e.g. emergency operations and a minimally invasive surgical approach. Additionally, as the ERAS protocol, an evidence-based care improvement process, is becoming more widespread throughout various fields in surgery^26,27^, the usefulness of postoperative coffee consumption within an ERAS context is a further relevant question to be addressed.

## Conclusions

Postoperative coffee consumption is effective for stimulating the recovery of gastrointestinal function after abdominal surgery. This intervention also reduces the length of hospital stay. The benefits appear to increase with an increased complexity of the surgical procedure.

## Electronic supplementary material

Supplementary information
