# Supplementary material for: Effect of postoperative coffee consumption on gastrointestinal function after abdominal surgery: A systematic review and meta-analysis of randomized controlled trials
Source: Sci Rep. 2018 Nov 26;8:17349. doi: 10.1038/s41598-018-35752-2 (PMC6255780; doi:10.1038/s41598-018-35752-2)
Supplement: Supplementary file 1 — Supplementary information [file 41598_2018_35752_MOESM1_ESM.pdf]

SUPPLEMENT INFORMATION

Effect of postoperative coffee consumption on gastrointestinal function after abdominal surgery: A systematic review and meta-analysis of randomized controlled trials

Authors

Nuntasiri Eamudomkarn[1], Chumnan Kietpeerakool[1],

Srinaree Kaewrudee[1], Nampet Jampathong[1],

Chetta Ngamjarus[2], Pisake Lumbiganon[1]

Institutions:

[1]Department of Obstetrics and Gynaecology, Faculty of Medicine

[2]Department of Epidemiology and Biostatistics, Faculty of Public Health

Khon Kaen University, Khon Kaen, Thailand

Supplement 1

MEDLINE search strategy (via OVID)

1. Caffeine/ or caffeine.mp. (26526)
2. exp Gastrointestinal Motility/
3. exp Intestinal Obstruction/ or exp Intestinal Pseudo-Obstruction/ or exp Ileus/ or exp Duodenal Obstruction/
4. exp Gastrointestinal Transit/
5. exp Peristalsis/
6. exp Auscultation/
7. exp Ileus/
8. exp Flatulence/
9. ileus$.tw.
10. bowel$ function$.tw.
11. (intestin$ adj5 pseudo-obstruct$).tw.
12. ((postoperative or post-operative) adj5 recover$).tw.
13. ((gastro-intestinal or gastrointestinal) adj5 function$).tw.
14. ((gastrointestinal or gastro-intestinal) adj5 dysmotilit$).tw.
15. ((gastrointestinal or gastro-intestinal) adj5 motilit$).tw.
16. ((gastrointestinal or gastro-intestinal) adj5 transit$).tw.
17. ((duoden$ or intestin$) adj5 obstruct$).tw.
18. (gut adj5 (motilit$ or dysmotilit$ or transit$)).tw.
19. (colorect$ or intestin$ or colectom$ or ileostom$ or colonic$ or gynecologic$ or gynaecolog$ or auscultation$ or peristalsis).tw.
20. ((postoperative or post-operative) adj5 ileus).tw.
21. (bowel adj5 (motlit$ or dysmotilit$ or transit$)).ti,ab.
22. (colon adj5 (motilit$ or dysmotilit$ or transit$)).ti,ab.
23. 2 or 3 or 4 or 5 or 6 or 7 or 8 or 9 or 10 or 11 or 12 or 13 or 14 or 15 or 16 or 17 or 18 or 19 or 20 or 21 or 22
24. 1 and 23
25. randomized controlled trial.pt.
26. controlled clinical trial.pt.
27. randomized.ab.
28. drug therapy.fs.
29. randomly.ab. (199356)
30. groups.ab. (1275406)
31. 25 or 26 or 27 or 28 or 29 or 30
32. 24 and 31

Table Supplement 1
Characteristics of included studies

**<u>Study 1</u>**<br>Title: Randomized clinical trial on the effect of coffee on postoperative ileus following elective colectomy<br>Authors: Muller SA, Rahbari NN, Schneider F, Warschkow R, Simon T, von Frankenberg M, et al.<br>Source: Br J Surg 2012;99(11):1530-8		
Methods	Randomized controlled trial	
Participants	Sample size: 80 patients<br>Exclusion criteria:<br>- Rectal resection was intended, a stoma was required or multivisceral resection was planned<br>- Hypersensitivity or distaste for coffee<br>- Expected lack of compliance<br>- Impaired mental state<br>- Intended colonic surgery was not performed	
Surgical procedure	Elective open or laparoscopic colonic resection for malignant or benign diseases	
Interventions	Intervention: three cups of coffee (100 ml) given at 08.00 12.00 and 16.00 hours), beginning on the morning after surgery<br>Control: warm water	
Outcomes	Primary outcome:<br>- Time to first defecation<br>Secondary outcomes:<br>- Time to first flatus<br>- Time to tolerance of solid food<br>- Need for additional laxatives<br>- Safety<br>- Length of hospital stay	
**Risk of bias assessment**		
Bias	Authors' judgement	Support for judgement
Random sequence generation (selection bias)	Low risk	Authors used randomizer software (SAS version 9.1) for random sequence generation
Allocation concealment (selection bias)	Low risk	Authors concealed allocation by sequentially numbered, opaque, sealed envelopes
Blinding of participants and personnel (performance bias)<br>All outcomes	High risk	No blinding
Blinding of outcome assessment (detection bias)<br>All outcomes	Unclear risk	No information available

Incomplete outcome data (attrition bias)<br>All outcomes	Low risk	All outcomes were measured during postoperative hospital stay
Selective reporting (reporting bias)	Low risk	All outcomes of interest reported
Other bias	Low risk	Study appeared free of other sources of bias
**<u>Study 2</u>**<br>Title: Effect of coffee on the length of postoperative ileus after elective laparoscopic left-sided colectomy: a randomized, prospective single-center study<br>Authors: Dulskas A, Klimovskij M, Vitkauskiene M, Samalavicius NE.<br>Source: Dis Colon Rectum 2015;58(11):1064-9		
Methods	Randomized controlled trial	
Participants	Sample size: 105 patients<br>Exclusion criteria:<br>- A stoma was required or multivisceral resection was planned<br>- Hypersensitivity or distaste for coffee<br>- Expected lack of compliance<br>- Impaired mental state	
Surgical procedure	Elective laparoscopic left-sided colonic resection for malignant diseases	
Interventions	Three groups<br>Group 1: three cups of coffee with caffeine daily (100 ml at 08.00 12.00 and 16.00 hours), beginning on the morning after surgery<br><br>Group 2: 3 cups of coffee without caffeine daily (100 ml at 08.00 12.00 and 16.00 hours), beginning on the morning after surgery<br><br>Group 3: warm water	
Outcomes	Primary outcome:<br>- Time to first defecation<br>Secondary outcomes:<br>- Time to first flatus<br>- Time to tolerance of solid food<br>- Length of hospital stay	
**Risk of bias**		
Bias	Authors' judgement	Support for judgement
Random sequence generation (selection bias)	Unclear risk	No data available

Allocation concealment (selection bias)	Low risk	Simple envelop method
Blinding of participants and personnel (performance bias) All outcomes	High risk	No blinding
Blinding of outcome assessment (detection bias) All outcomes	Low risk	Recorded by a nurse blinded to the intervention allocated
Incomplete outcome data (attrition bias) All outcomes	Low risk	All outcomes were measured during postoperative hospital stay
Selective reporting (reporting bias)	Low risk	All outcomes of interest reported
Other bias	Low risk	Study appeared free of other sources of bias

**<u>Study 3</u>**<br>Title: Effects of coffee consumption on gut recovery after surgery of gynecological cancer patients: a randomized controlled trial<br>Authors: Gungorduk K, Ozdemir IA, Gungorduk O, Gulseren V, Gokcu M, Sanci M.<br>Source: Am J Obstet Gynecol 2017;216(2):145 e1- e7	
Methods	Randomized controlled trial
Participants	Sample size: 118 patients<br>Exclusion criteria:<br>- Hypersensitivity or allergy to caffeine/ coffee<br>- Thyroid disease<br>- Inflammatory bowel disease<br>- Compromised liver function<br>- Clinically significant cardiac arrhythmia<br>- Chronic constipation (defined as 2 bowel movements per week)<br>- History of abdominal bowel surgery<br>- Previous abdominal irradiation<br>- Previous neoadjuvant chemotherapy or hyperthermic intraperitoneal chemotherapy<br>- A need for intensive care for >24 hours postoperatively<br>- A need for nasogastric tube drainage beyond the first postoperative morning<br>- A bowel anastomosis<br>- The use of an upper abdominal multivisceral surgical approach for debulking surgery
Surgical procedure	Comprehensive staging surgery (abdominal hysterectomy and systematic pelvic and paraaortic lymphadenectomy) in whom diagnosis of cervical, endometrial or ovarian cancer
Interventions	Intervention: three cups of caffeinated coffee daily (100 ml at 10.00 15.00 and 19.00 hours), beginning on the morning after surgery

	Control: no intervention	
Outcomes	Primary outcome:<br>- Time to first flatus<br>Secondary outcomes:<br>- Time to first defecation<br>- Time to first bowel sound<br>- Time to tolerance of solid food<br>- Side effects of coffee intake<br>- Postoperative paralytic ileus type and rate<br>- Length of hospital stay	
**Risk of bias**		
Bias	Authors' judgement	Support for judgement
Random sequence generation (selection bias)	Low risk	Authors used computer-generated code for running a block randomization
Allocation concealment (selection bias)	Low risk	Simple envelop method
Blinding of participants and personnel (performance bias)<br>All outcomes	High risk	No blinding
Blinding of outcome assessment (detection bias)<br>All outcomes	Low risk	The evaluator was blinded to the study allocation
Incomplete outcome data (attrition bias)<br>All outcomes	Low risk	All outcomes were measured during postoperative hospital stay
Selective reporting (reporting bias)	Low risk	All outcomes of interest reported
Other bias	Low risk	Study appeared free of other sources of bias
**Study 4**<br>Title: Effect of gum chewing and coffee consumption on intestinal motility in caesarean sections.<br>Authors: Göymen A, Şimşek Y, Özkaplan ŞE, Özdurak Hİ, Akpak YK, Semiz A, et al.<br>Source: J Clin Anal Med. 2016.DOI: 10.4328/JCAM.		
Methods	Randomized controlled trial	
Participants	Sample size: 100 pregnant patients<br>Exclusion criteria:<br>- Preterm labour<br>- Multiple pregnancies<br>- Premature rupture of membranes<br>- Emergency caesarean section	

	- Body temperature above 38°C<br>- Severe anaemia<br>- History of abdominal surgery other than caesarean section<br>- Allergic reaction to the agents used<br>- Inability to chew gum	
Surgical procedure	Elective caesarean section in pregnant patients with completion of gestational week 37	
Interventions	Four groups<br>Group 1: sugar-free gum at 4 hours intervals after postoperative hour 2 until first defecation<br><br>Group 2: 100 ml coffee at 4 hours intervals beginning from postoperative hour 2 until defecation for three times a day<br><br>Group 3: 100 ml hot water at 4 hours intervals beginning from postoperative hour 2 until defecation for three times a day<br><br>Group 4: No intervention	
Outcomes	- Time to first bowel sound<br>- Time to first flatus<br>- Time to first defecation	
**Risk of bias**		
Bias	Authors' judgement	Support for judgement
Random sequence generation (selection bias)	Low risk	Authors used a simple randomization method with the aid of a computer
Allocation concealment (selection bias)	Unclear risk	No information provided
Blinding of participants and personnel (performance bias)<br>All outcomes	High risk	No blinding
Blinding of outcome assessment (detection bias)<br>All outcomes	Unclear risk	The research assistant evaluated outcome, but no information about blinding the assistant available
Incomplete outcome data (attrition bias)<br>All outcomes	Low risk	All outcomes were measured during postoperative hospital stay
Selective reporting (reporting bias)	Low risk	All outcomes of interest reported
Other bias	Low risk	Study appeared free of other sources of bias

**Study 5**<br>Title: Does coffee affect the bowel function after caesarean section?<br>Authors: Rabiepoor S, Yas A, Navaei J, Khalkhali HR.<br>Source: Eur J Obstet Gynecol Reprod Biol 2018;220:96-9		
Methods	Randomized controlled trial	
Participants	Sample size: 100 patients<br>Exclusion criteria:<br>- Gastrointestinal complications<br>- Respiratory problems and infections that required medicational interventions<br>- Surgery longer than 90 min	
Surgical procedure	Elective caesarean section	
Interventions	Intervention: three cups of 100cc sugar-free coffee at 8, 12 and 20 hr after the surgery<br>Control: 100cc hot water at the same intervals	
Outcomes	- Time to first bowel sound<br>- Time to first flatus<br>- Time to first defecation<br>- Length of hospital stay	
**Risk of bias**		
Bias	Authors' judgement	Support for judgement
Random sequence generation (selection bias)	Unclear risk	Insufficient information about the sequence generation process
Allocation concealment (selection bias)	Low risk	Simple envelop method
Blinding of participants and personnel (performance bias)<br>All outcomes	High risk	No blinding
Blinding of outcome assessment (detection bias)<br>All outcomes	Unclear risk	No information available
Incomplete outcome data (attrition bias)<br>All outcomes	Low risk	All outcomes were measured during postoperative hospital stay
Selective reporting (reporting bias)	Low risk	All outcomes of interest reported
Other bias	Low risk	Study appeared free of other sources of bias

**Study 6**<br>Title: Immediate postoperative coffee consumption stimulates early gut recovery after cesarean section<br>Author: Mohamed Farag El Sherbany<br>Source: www.sematicscholar.org (available at https://pdfs.semanticscholar.org/8b79/0eb67f979bea8affae03fe5010167ae166c0.pdf, Accessed October 2, 2018)		
Methods	Randomized controlled trial	
Participants	Sample size: 210 patients<br>Exclusion criteria:<br>- Pre-existing bowel disease<br>- History of prior abdominal bowel surgery<br>- History of prior upper or lower abdominal exploration<br>- Clinically significant cardiac arrhythmia<br>- Chronic constipation (defined as ≤ 2 bowel movements per week)<br>- Any known hypersensitivity or allergy to caffeine/ coffee<br>- Need for intensive care for > 24 hours<br>- Need for nasogastric tube drainage or need for surgery other than CS as cesarean hysterectomy or extensive intra-abdominal surgery as a result of intraoperative complications<br>- Distaste of coffee	
Surgical procedure	Elective caesarean section	
Interventions	Intervention: four cups of 100 ml of coffee, every 4 hours during daytime, beginning immediate postoperatively as soon as the patient could do and continued up to first bowel motion either flatus or defecation<br>Control: no intervention	
Outcomes	Primary outcome:<br>- Time to first flatus<br>Secondary outcomes:<br>- Time to first defecation<br>- Time to first bowel sound<br>- Time to tolerance of solid food<br>- Side effects of coffee intake<br>- Length of hospital stay	
**Risk of bias**		
Bias	Authors' judgement	Support for judgement
Random sequence generation (selection bias)	Low risk	Authors used computer – generated code running a blocked randomization

Allocation concealment (selection bias)	Low risk	Authors concealed allocation by sequentially numbered, opaque, sealed envelopes
Blinding of participants and personnel (performance bias)<br>All outcomes	High risk	No blinding
Blinding of outcome assessment (detection bias)<br>All outcomes	Unclear risk	No information available
Incomplete outcome data (attrition bias)<br>All outcomes	Low risk	All outcomes were measured during postoperative hospital stay
Selective reporting (reporting bias)	Low risk	All outcomes of interest reported
Other bias	Low risk	Study appeared free of other sources of bias

Table Supplement 2
Characteristics of ongoing studies

**Ongoing study 1**<br>**Contact author: Fernando Aguila**	
Methods	Randomized controlled trial
Participants	Sample size: 120 patients<br>Exclusion criteria:<br>- Patient with atrial fibrillation who is considered a non-coffee drinker (drinks coffee less than 3 days per week over the last 4 weeks)<br>- Patients undergoing colon resection without removal of any portion of small bowel
Surgical procedure	Elective, urgent or emergent small bowel resection
Interventions	Intervention: 100 ml of coffee administered 3 times per day until return to bowel function has been established<br>Control: 100 cc of warm water administered 3 times per day until return to bowel function has been established
Outcomes	Primary outcome:<br>- Time to removal of nasogastric tube<br>Secondary outcomes:<br>- Time to return of bowel function (first flatus or bowel movement)<br>- Length of hospital stay
The most update status	Recruiting participants
Trial registration number	ClinicalTrials.gov Identifier: NCT03143621

**Ongoing study 2**<br>**Contact author: Gehrer Simone**	
Methods	RCT
Participants	136 patients<br>Exclusion criteria:<br>- Participation in other studies<br>- Additional small bowel anastomosis<br>- Need for extended adhesiolysis<br>- Need for a stoma (e.g. protective ileostomy)<br>- Emergency operation with diffuse peritonitis or preexisting ileus

	- Preoperative radiation<br>- Known hypersensitivity or allergy to coffee<br>- Expected lack of compliance<br>- Impaired mental state or language problems
Surgical procedure	Elective laparoscopic or open colorectal surgery due to benign or malignant colorectal disease, which need a large bowel resection with primary anastomosis
Interventions	2 groups<br>Treatment: coffee<br>Control: water/tea (excluding black tea)
Outcomes	Time to first defecation
The most update status	Completed recruitment
Trial registration number	ClinicalTrials.gov Identifier: NCT02469441
**Ongoing study 3**<br>**University of Massachusetts**	
Methods	RCT
Participants	44 patients<br>Exclusion criteria:<br>- Total colectomy<br>- Colostomy<br>- Ileostomy<br>- Reversal of a stoma or synchronous resection<br>- Complete small or large bowel obstruction<br>- Scheduled to receive other treatments or techniques to reduce ileus (epidural anesthetic techniques)<br>- Nasogastric tube for any length of time in the post-op period.
Surgical procedure	Laparoscopic or laparotomic elective partial bowel resection with primary anastomosis for either cancer or benign disease
Interventions	2 groups<br>Treatment: 8 oz. of caffeinated coffee/breakfast &noon meal<br>Control: no intervention
Outcomes	- Time to first flatus<br>- Time to first bowel sound<br>- Time to tolerance of solid food

The most update status	Completed recruitment
Trial registration number	ClinicalTrials.gov Identifier: NCT01130675
**Ongoing study 4**<br>**Contact author: Yosef Nasseri**	
Methods	RCT
Participants	300 patients<br>Exclusion criteria:<br>- Patients with a history of prior intestinal surgery, excluding appendectomy.<br>- Patients who require an ostomy during their elective colorectal surgery.<br>- Patients who require postoperative ventilation, pressures, or ICU stay.<br>- Those who are mentally incompetent, unable, or unwilling to provide informed consent or comply with study procedures.<br>- American Society of Anesthesiologist (ASA) class IV or V.<br>- Those with a history of carcinomatosis.<br>- Those with a history of radiation enteritis.<br>- Women who are pregnant.<br>- Patients who have a history of epilepsy.<br>- Patients with prior cardiovascular disorders including uncontrolled hypertension, prior myocardial infarction, or heart failure.<br>- Patients with peptic ulcers.<br>- Patients with glaucoma.<br>- Non-English Speakers
Surgical procedure	Small and/or large partial bowel resection via laparotomy or laparoscopy with primary anastomosis
Interventions	Three groups<br>**Group A**: Regular coffee<br>4oz cup of coffee, three times daily (at 8:00, 12:00, and 16:00 hours)<br>Duration of experimental treatment will last until first flatus or bowel movement or 7 days, whichever comes first.<br>**Group B:** Decaffeinated coffee<br>4oz cup of decaffeinated coffee, three times daily (at 8:00, 12:00, and 16:00 hours)<br>Duration of experimental treatment will last until first flatus or

	bowel movement or 7 days, whichever comes first.<br>**<u>Group C:</u>** Warm water<br>4oz cup of warm water, three times daily (at 8:00, 12:00, and 16:00 hours)<br>Duration of experimental treatment will last until first flatus or bowel movement or 7 days, whichever comes first.
Outcomes	Primary outcome:<br>- Time to first flatus and/or bowel movement<br>Secondary outcomes:<br>- Length of hospital stay<br>- Vomiting<br>- Nasogastric tube insertion<br>- Anastomotic leakage<br>- Wound infection<br>- Abscesses
The most update status	Unknown status of study
Trial registration number	ClinicalTrials.gov Identifier: NCT02639728
